# Treatment process for capillary hemangioma

**Published:** 2014-07-20

**Authors:** NB Mirbehbahani, A Rashidbaghan

**Affiliations:** 1 Golestan University of Medical Sciences, Taleghani hospital, Janbazan Boulevard, Gorgan, Iran.; 2 Golestan University of Medical Sciences, Taleghani hospital, Janbazan boulevard, Gorgan, Iran.

**Keywords:** hemangioma, propranolol, interferon alpha

## Abstract

**Background:**

Hemangiomas, usually, present at the first few months of life and are the most common benign tumor in children. There are various therapeutic methods for hemangioma. Capillary hemangioma is a type of hemangiomas.

The steps of treatment of a child with capillary hemangioma in Taleghani Hospital of Gorgan, Iran, are reported.

**Case report:**

In this report, it is described an 18-month-old child with capillary hemangioma on the right side of face. She was presented to the hematologic clinic of Taleghani Hospital of Gorgan. Three drugs, including prednisolon, propranolol and interferon alpha-2b (IFN-α-2b), were used for treating this patient. At the end of treatment, good results were obtained. After that, laser therapy was performed for fading the lesions.

**Conclusion:**

Prescription of drug was our first choice for treating capillary hemangioma and it had a positive result without any complications. We used propranolol and IFN-α-2b for treating capillary hemangioma because of their better effect on this patient.

## Introduction

Vascular lesions are classified as hemangiomas or vascular malformations (1). Hemangiomas, usually, present at the first few months of life and are the most common benign tumor in children. Mostly they are appeared on the skin and mucous membranes of the head and neck region and they are classified based on their location as superficial, deep, or mixed (1,2). About 1 to 3% of all infants are born with hemangiomas (3), occurring in 4 to 12% of all Caucasion (4). The ratio of affected females to males is 3:1 (5).

Hemangiomas classified into capillary, cavernous and capillary cavernous based on their depth in the dermis and subcutaneous fat (2). Ulceration, bleeding, scarring and infection are complications of hemangiomas as well as dysfunction in vision, respiration, hearing or feeding (5). Various therapeutic options for treatment of hemangiomas include observation for spontaneous remission, and using topical, intralesional and systemic corticosteroids, cryosurgery, interferon, radiation, embolization, and laser therapy as [CO2 laser PDL, KTP and ND-YAG (6). We describe a child who was treated successfully for hemangiomas with prednisolone, propranolol and interferon alpha-2b (INF-α-2b). 

## Case report

**Figure 1:**
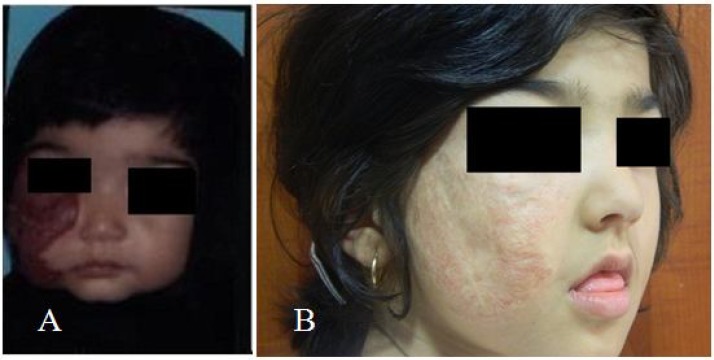
A patient with right-side capillary hemangioma

An 18-month-old girl with capillary hemangioma on the right side of face was admitted to the hematologic clinic of Taleghani Hospital of Gorgan, Iran (figure 1, A). The diagnosis was clinical. The lesion was unilateral, red, coetaneous, lumpy and raised nodular that blenches with pressure. The capillary tumor on her face had been appeared from birth and progressed gradually. Laboratory investigation, inclouding compelet blood count was normal and brain CT scan did not show any lesions. Liver and spleen hemangioma were not found in abdominal ultrasound. At first year of age, oral treatment was initiated with prednisolone (2.5 mg/kg in two divided doses daily) for 2 months without treating effect. Treatment with propranolol (2 mg/kg in two divided two doses daily) was begun with prednisolone for 2 months. The child improved during combined therapy. Monitoring of vital signs and blood pressure was checked weekly. Unfortunately, despite the good response to treatment, the combined therapy was stopped because the patient gave up receiving oral medicine. After 2 months, the treatment was started again with interferon alpha-2b at a dose of 3 million units/m2 3 times in a week for three months, subcutaneously. Then IFN-α-2b therapy continued with the same dose as 2 times in a week for three months. During subsequent 3 months, the times of injection reduced to one time per week and finally during latest 6 months, IFN-α-2b was received monthly. During treatment with IFN-α-2b, evaluation of liver enzyme concentration, neurological symptoms and CBC (Cell Blood Count) was conducted. At this period, good results were obtained and significant reduction was observed in hemangioma size (figure 1, B). At the end of treatment, 3 medical lasers were used for removing skin lesions. The laser therapy caused to fade these lesions. 

## Discussion

Capillary or strawberry hemangioma involves only the dermis (2) is the most frequent benign eyelid and orbitary tumor in children. Depending on its depth, capillary hemangioma can cause complications such as anisometropia, strabismus or amblyopia (7).

: A) before beginning treatment; B) after treatment.

Foi this patient admitted to the hematologic clinic of Taleghani Hospital of Gorgan, we used 3 methods for treating capillary hemangioma inclouding prednisolon (one of steroids), propranolol and IFN-α-2b, respectively.

The response rate to steroids was variable, and complications were common. Weber et al., 1990, treated 11 children with prednisone with a dose of 3 to 8 mg/kg/day. The results showed that only 2 (18%) patients were cured, and there was clear failure of therapy in 4 (36%) persons. Hypertension developed in 5 (45%) of patients. Finally, all children were cured, with minimal morbidity (1). Enjolras, 1990, found various responses to steroids in a study on 25 children with alarming hemangiomas, so that the response was excellent in 30% of patients, 30% of them had no response and treatment was slow and doubtful in remaining 40%. Also, no evidence was found to show lack of response in steroid therapy. (1). Treatment with prednisolone did not have positive effect on our case. It was selected as the first choice of therapy because it was the cheapest and most effective available medicine for treating hemangioma in that condition. 

Propranolol was the second option for treatment of our patient. It blocks β1 and β2 receptors but the mechanism of its action is not clear. It seems beta-blocker induce apoptosis by antagonizing Glut-1 receptors or act through other pathways to inhibit growth of the hemangioma of infancy (8). Propranolol had a positive effect on hemangioma in our study. There are some studies that are in agreement with our study. Lowly et al., 2009, reported two patients with hemangima on the eyelid. They were treated with propranolol and the therapy was satisfactory for both of them (8). Aletaha et al., 2012, treated 4 children aged 3 months to 5 years with periocular and orbital hemangioma of infancy in Iran with propranolol. Significant improvement was noted for all patients in the first 2 months of treatment and continued slowly during the follow-up without any serious complications (9). Talaate, 2012, treated 50 infants with hemangioma by oral propranolol and they observed changes in color, softening and size of hemangiomas. Collectively, high efficacy and tolerance of propranolol treatment has been elicited (10). Salazar-Murillo, 2012, introduced an infant with capillary hemangioma on the left side of the face that was treated by propranolol. This therapy was effective in that study (7). The expense of propranolol is low as well as its extraordinary effect and if propranolol therapy is continued, we achieved a better result. Unfortunately, we had to use IFN-α-2b for treating hemangioma because of non-coopration of patient for monitoring of blood pressure and frequent returning. 

IFN-α is a family of homologous, species-specific proteins that acts as complex anti-viral, anti-neoplastic, and immunomodulating factors (11). The exact mechanism of IFN action is unknown, but it may act as an angiogenesis inhibitor directly, because it can inhibit both endothelial cell and fibroblast proliferation and production of endothelial prostaglandins. Also its effects may be are indirectly by inhibiting angiogenic stimulus as inhibiting the effects of specific growth factors on the proliferation of endothelial cells, smooth muscle cells, or fibroblasts, decreasing the production of collagen, enhancing the production or release of endothelial cell prostacyclin (1). Usually, IFN is used for life or sight-threatening capillary hemangiomas (11). In 1980, Brouty-Boye and Zetter reported that IFN inhibited capillary endothelial cell migration in vitro and it was the starting point of treating hemangiomas by IFN. In 1987, Friesel reported that IFN-τ inhibited endothelial cell proliferation in vitro (1). Sidkey and Borden, also in 1987, reported that IFN inhibited tumor-induced angiogenesis in vivo in a murine model. In this regard, White et al., 1989, obtained regression of pulmonary hemangiomatosis in a 12-year-old boy treated with IFN-α-2a. This treatment resulted in improving the exertional dyspnea and clubbing in patient and also normalizing the pulmonary function tests and pulmonary angiogram. In that same year, Orchard et al. reported extraordinary response to IFN-a-2b in two infants, one with uncontrollable Kasabach-Merritt syndrome and one with a large facial hemangioma. Ezekowitz et al., 1992, reported the results of a clinical trial study on treatment of hemangiomas with IFN-α-2a on 20 patients in Boston Children's Hospital. The age range was 3 weeks to 2 years. Four had Kasabach-Merritt syndrome; ten had head, neck, or airway lesions; three had periorbital lesions; and three had lesions in other locations. Regression of the hemangioma was 50% or more during 7.8 months of treatment in 18 of 20 patients. One of patients died from Kasabach-Merritt syndrome. In three of patients who had large hemangiomas and did not have any responses to conventional therapies, the hemangioma stabilized after seven days of treatment with IFN-α-2a alone (1). McArthur, 1995, treated 5 patients with massive hemangoimas of the head and neck area by IFN-α-2a at the University of California. Use of this drug was successful in those patients (3). Rickette, 1994, studied 4 infants and one child with complex hemangioma for effect of IFN-α-2a. Although, two patients experienced minor complications that were managed easily, but the treatment by this medicine was beneficial (1). IFN-α-2b had a positive effect on our patient the same as other studies. Also, Teske demonstrated satisfactory effect of IFN-α for treating capillary hemngioma in 1994. Patients in that study were two female infants with capillary hemangioma (12). Because IFN-α-2b is more expensive than previous drugs and must be injected, it was final option for treating our patient in our treatment method.

## Conclusion

From various methods for treating hemangioma, using of drug was our first choice and it has positive result in treating our patient. Three drugs were used subsequently without any complications. According to our observation propranolol and IFN-α-2b are suggested for treating capillary hemangioma.
